# Awareness of Korean Dental Professionals about the Need for Autonomy in Dental Hygiene Practice

**Published:** 2018-09

**Authors:** Joo-Young LEE, Gyeong-Soon HAN

**Affiliations:** 1. Dept. of Preventive Dentistry and Public Oral Health, Brain Korea 21 PLUS Project, Yonsei, University College of Dentistry, 50-1 Yonsei-ro, Seodaemun-gu, Seoul, Republic of Korea; 2. Dept. of Dental Hygiene, College of Health Science, Gachon University, 191 Hambangmoe-ro, Yeonsu-gu, Incheon, Republic of Korea

## Dear Editor-in-Chief

As of 2017, the number of registered dental hygienists completed the requirements of dental hygiene education in Korea are 75883. Course-work in dental hygiene is offered by 82 colleges-54 with three-year programs and 28 with four-year programs. The doctoral education program in dental hygiene was approved for the first time in Korea in 2013. It would pave the way for improving dental hygiene studies, a challenge to be met with the opening of the Korean Institute of Dental Hygiene Education and Evaluation.

Independent dental hygiene practices that are not overly regulated under the legal system have been adopted in many other countries, including the United States, Canada, and Europe (1). In Korea, on the other hand, it expresses education and preventive measures, but the work of clinical assistance is the most and the autonomy of work is low and limited. The Korean dental hygiene curriculum features a program promoting autonomous practice that includes periodontal treatment. The dental hygiene judged to have sufficient competence because they dental hygienists are greater efficient than dentists in performing infection control, conducting follow-ups to medical findings, analyzing patients’ medical histories, and completing periodontal and mucosal charting (2).

Restrictive dental hygiene regulations should be amended reasonably based on socioeconomic effects. Providing the best dental treatments by the most cost-effective means will ensure that dental management is economical, benefiting both dental professionals and patients (2). The aim of this study was to determine attitudes of dentists and dental hygienists toward supporting independent dental hygiene practices and improving regulations affecting dental hygienists.

Accordingly, this survey was conducted from Mar to May 2014. All participating individuals signed informed consent forms before they completed the self-report questionnaire. Questionnaires were sent to dentists and dental hygienists asked to complete and return them. Survey data were collected from 158 dentists and 258 dental hygienists (Head DH: 97, General DH: 161) completed the questionnaire in its entirety. The 5-point Likert scale (1-*strongly disagree* to 5-*strongly agree*) employed in the questionnaire was used to evaluate the following (Cronbach’s alpha value: CAV):

1. Agreement of autonomy in dental hygiene practice: assessment, dental hygiene diagnosis, planning, implementation, evaluation (CAV: 0.908)
2. Benefits of autonomy in dental hygiene practice: work efficiency, patient management, satisfaction of staff, hospital image (CAV: 0.870)
3. Obstructive factors of autonomy in dental hygiene practice: dental settings, capability of DH, lack of necessity (CAV: 0.829)

The collected data were analyzed using SPSS WIN 21.0 (IBM Co., Armonk, NY, USA). Comparisons among groups were performed by a one-way analysis of variance followed by Duncan’s Test (*P*<0.05). The results were presented by ladder graphs to depict rankings of perceptions among groups.

**Fig. 1:**
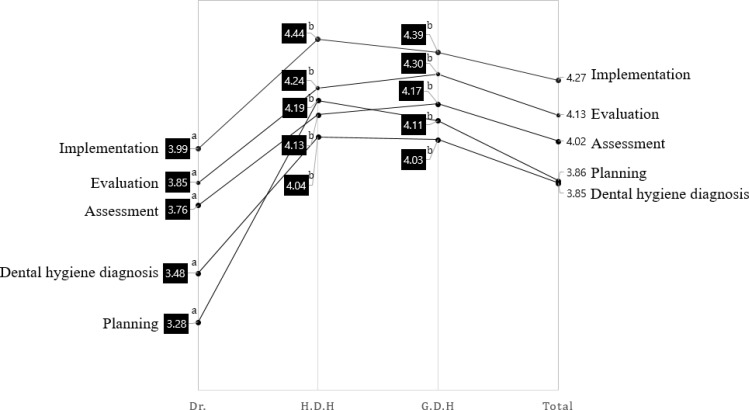
Ranking of Autonomy in dental hygiene practice among dental professional Dr.: Dentist, H.D.H: Head Dental Hygienist, G.D.H: General Dental Hygienist, Total: Dental professional. ^a,b^ The same characters were not significant by Duncan comparison at α=0.05

Figure 1 shows a comparison of rankings regarding autonomy in dental hygiene practices (five steps) among dental professionals.

The highest ranked response was implementation (4.27 points) and the lowest ranked responses were planning (3.86 points) and dental hygiene diagnosis (3.85 points). There were statistically significant differences in the mean responses of dentists and dental hygienists for all statements (*P*<0.001, Tukey’s HSD test). The difference between the group was significantly higher for the dental hygienist than for the dentist (*P*<0.001), which indicates that there is a clear difference between the two job categories for the dental hygienists work.

Regarding fieldwork on dental hygiene diagnosis, most Australian dental hygienists showed higher agreement (above 4 points on the Likert scale) than Korean hygienists (3). Korean dental hygienists are required to perform under the direct supervision of dentists in most settings. To provide high-quality dental care, dental hygienists should be legally permitted to perform their work independently.

**Fig. 2:**
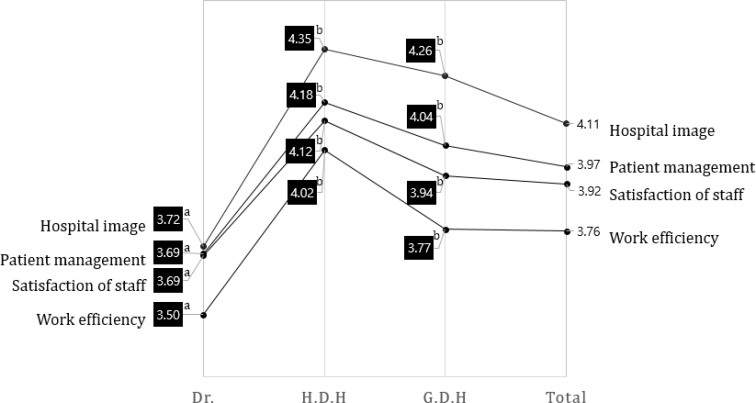
Ranking of Beneficence of Autonomy in dental hygiene practice among dental professional Dr.: Dentist, H.D.H: Head Dental Hygienist, G.D.H: General Dental Hygienist, Total: Dental professional. ^a,b^ The same characters were not significant by Duncan comparison at α=0.05

All dental professionals seemed to follow a similar pattern related to perspectives on autonomy in dental hygiene practice (Fig. 2). The most beneficial factor was judged as “improving hospital image” (4.11 points) and “improvement work efficiency” (3.76 points) was the lowest. All dental hygienists were significantly higher than dentists (*P*<0.001). According to a Scottish report, tasks performed by a hygienist increase a dentist’s daily income by 33% (4).

**Fig. 3:**
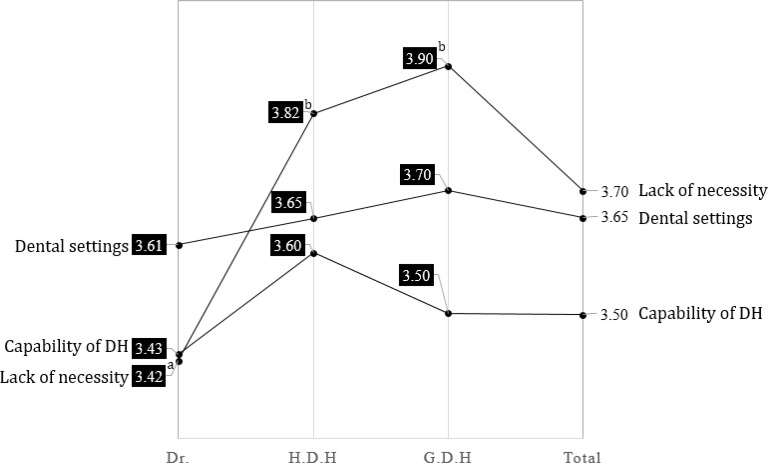
Ranking of Obstructive factors of Autonomy in dental hygiene practice among dental professional Dr.: Dentist, H.D.H: Head Dental Hygienist, G.D.H: General Dental Hygienist, Total: Dental professional. ^a,b^ The same characters was not significant by Duncan comparison at α=0.05

In relation to this, HDH showed a confidence of more than 4.0 points for autonomous work in all areas and showed that the dentist had less than 4.0 points in all areas. It is necessary to consider the solution carefully. Major barriers to autonomy in dental practice were perceived as a lack of necessity and insufficient dental settings for dental hygiene groups (Fig. 3, *P*<0.001). Therefore, changes in educational curricula and international practices of dental hygienists are needed to support a paradigm shift regarding the role of the dental hygienist. Multidimensional cooperation with Korean dental associations, committees of dental hygiene professors, and relevant administrative agencies are also needed.
